# A Trust Management Model for IoT Devices and Services Based on the Multi-Criteria Decision-Making Approach and Deep Long Short-Term Memory Technique

**DOI:** 10.3390/s22020634

**Published:** 2022-01-14

**Authors:** Yara Alghofaili, Murad A. Rassam

**Affiliations:** 1 Department of Information Technology, College of Computer, Qassim University, Qassim 52571, Saudi Arabia; 411207305@qu.edu.sa; 2 Faculty of Engineering and Information Technology, Taiz University, Taiz 6803, Yemen

**Keywords:** trust management, Internet of Things services, deep long short-term memory, multi-criteria decision-making, simple multi-attribute rating

## Abstract

Recently, Internet of Things (IoT) technology has emerged in many aspects of life, such as transportation, healthcare, and even education. IoT technology incorporates several tasks to achieve the goals for which it was developed through smart services. These services are intelligent activities that allow devices to interact with the physical world to provide suitable services to users anytime and anywhere. However, the remarkable advancement of this technology has increased the number and the mechanisms of attacks. Attackers often take advantage of the IoTs’ heterogeneity to cause trust problems and manipulate the behavior to delude devices’ reliability and the service provided through it. Consequently, trust is one of the security challenges that threatens IoT smart services. Trust management techniques have been widely used to identify untrusted behavior and isolate untrusted objects over the past few years. However, these techniques still have many limitations like ineffectiveness when dealing with a large amount of data and continuously changing behaviors. Therefore, this paper proposes a model for trust management in IoT devices and services based on the simple multi-attribute rating technique (SMART) and long short-term memory (LSTM) algorithm. The SMART is used for calculating the trust value, while LSTM is used for identifying changes in the behavior based on the trust threshold. The effectiveness of the proposed model is evaluated using accuracy, loss rate, precision, recall, and F-measure on different data samples with different sizes. Comparisons with existing deep learning and machine learning models show superior performance with a different number of iterations. With 100 iterations, the proposed model achieved 99.87% and 99.76% of accuracy and F-measure, respectively.

## 1. Introduction

**Figure 1:**
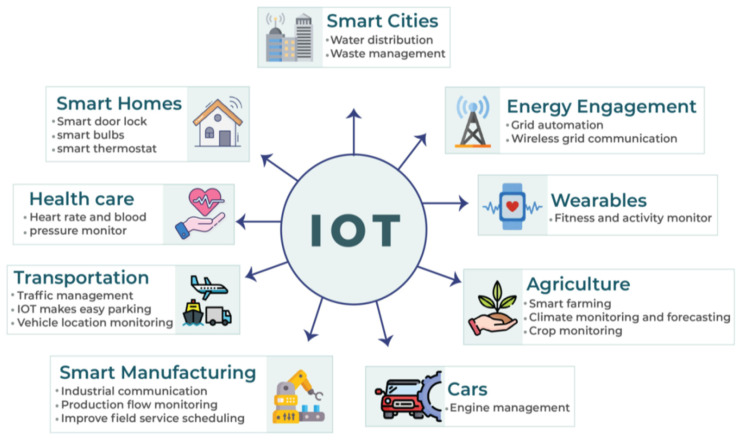
IoT applications and services.

The rationale behind the Internet of Things (IoT) paradigm was proposed way back in the 1980s with the idea of ubiquitous computing [1]. However, the term IoT received significant attention after the study by [2]. IoT’s objective is to incorporate technology into everyday life. Today, the modern IoT environment includes networking and social interactions between physical and cyber components [3]. IoT infrastructure helps run several innovative services (called IoT services) on various platforms, where a large number of heterogeneous devices work together to achieve a common objective [4]. IoT services are used to perform the actual sensing or actuation tasks [5]. In recent years, increasing focus has been given to IoT services in various fields, as seen in Figure 1, which can play an essential role in facilitating humans’ daily life [1].

The features that IoT has, such as the diversity of the shared data, dynamicity, and the heterogeneity of devices, bring an entirely new challenge to IoT services and devices. These challenges are mostly solved by considering the security issues in general without evaluating the subjective risks between IoT entities and services [6]. It might result in catastrophic harm and unknown dangers if the information is used for malicious purposes. The principle of trust in IoT can therefore be regarded as a critical feature for establishing trustworthy and reliable service provisioning between different objects [7]. As a result, trust has become one of the major requirements to achieving security.

Trust is an abstract notion, with varying definitions depending on both participants and situations, and informed by measurable and non-measurable variables [7]. This fact indicates that trust is a very complex concept that refers to other factors, such as the ability, strength, reliability, goodness, availability, or other characteristics of an object [8]. Trust management is, therefore, more challenging than security itself, especially in the emerging information technology field, such as IoT [9]. The concept “trust in IoT” refers to the examination of the behavior of devices linked to the same network. The trust connection between two devices influences the future behavior of their interactions. When devices trust each other, they prefer to share services and resources to some degree. Trust management enables the computation and analysis of trust among devices in order to make appropriate decisions for establishing efficient and reliable communication among devices.

Trust management is considered to be a viable solution for IoT trust issues. Such solutions have been used to optimize protection, support decision-making processes, identify untrusted behavior, isolate untrusted objects, and redirect functionality to trusted zones [10]. Various approaches, such as [11,12,13,14], have been developed by researchers as solutions to trust issues. However, these solutions are still unable to fully address trust issues and face numerous challenges, such as a lack of effectiveness when dealing with large amounts of data and constantly changing behaviors, high energy utilization, difficulty in quantifying uncertainty for untrusted behaviors, choosing the optimal trust model components, and dealing with IoT’s dynamic nature and heterogeneity.

Aimed at the former issues, this paper proposes a trust management model for IoT devices and services that takes leverage from multi-criteria decision-making and deep learning techniques. This model can identify suspicious activities and take appropriate actions, such as isolating untrusted entities and redirecting IoT functionality to trustworthy zones. The main contributions of this paper include: (1) creating a new dataset by extracting more features from real packet captures and patches that already exist in the literature; (2) calculating the trust value using the simple multi-attribute rating technique (SMART), which determines the value of the trust depending on the node information only to reduce the risk of threats that result from wrong recommendations. Additionally, it reduces the energy computation that makes the algorithm lightweight; and (3) Developing an intelligent solution based on the long short-term memory (LSTM) technique that counters the continuous change in behaviors as well as being compatible with big data to ensure stockholders benefit from these integral and available services of IoTs.

The rest of this paper is organized as follows: a background on trust management is presented in Section 2. The related works are investigated and discussed in Section 3. The proposed model is described in Section 4. The experimental investigation, result analysis, and evaluation of the proposed model are reported in Section 5, Section 6 and Section 7 reports the comparison results with existing models and Section 8 concludes the paper and suggests some future research directions.

## 2. Trust Management Principles and Terminologies

**Figure 2:**
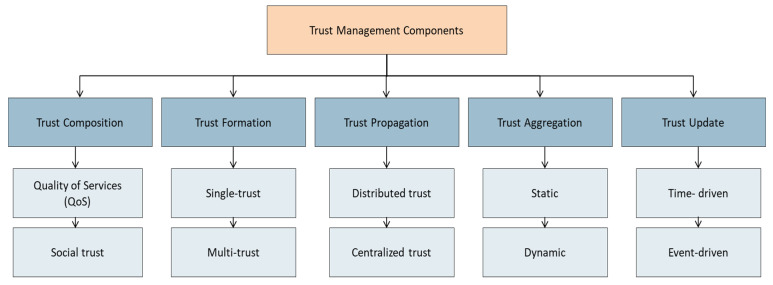
Trust management model components.

In this section, the main components of building a trust management model are introduced. These components are referred to as computational trust modules and are used to quantify and evaluate entity attributes, such as integrity, reliability, honesty, and others, to estimate the value of trust [6]. Figure 2 shows the five components, and the subsequent sections describes these components.

### 2.1. Trust Composition

This refers to the components that are considered in trust computation and it involves two major modules namely: quality of service (QoS) trust and social trust [8].

(1) QoS refers to the expectation of an IoT entity to provide superior quality in its functionalities. QoS trust utilizes some trust properties, such as competence, reliability, task completion capability, and cooperativeness, to measure the value of trust [8].
(2) Social trust refers to a social relationship among IoT entity owners. Social relationship trust is used to assess the IoT entity to evaluate whether it is trustworthy or not. Besides, social trust utilizes trust properties, such as honesty, centrality, intimacy, privacy, and connectivity, to measure trust values [8].

### 2.2. Trust Formation

This refers to whether trust computation is based on either one trust attribute (single-trust) or the use of multiple attributes (multi-trust). Besides, these components are chiefly concerned with what weights are put on QoS and social trust attributes from trust [8].

### 2.3. Trust Propagation

Trust propagation refers to the way of propagating trust information to other entities. Under this form of propagation [6], two main schemes can be identified:

1. Distributed trust refers to IoT entities autonomously propagating trust and observations to other IoT entities they interact with or encounter without the necessity for a centralized entity [6].
2. Centralized trust requires the presence of centralized entities. It can exist as either a virtual trust service or a physical cloud that is implemented by IoT devices [6].

### 2.4. Trust Aggregation

This refers to the most appropriate method of aggregating trust information, which is then evaluated by the entity itself (direct evaluation) or by other entities (indirect evaluation) [8]. This component aggregates information using weights, which might be static or dynamic. The static is calculated in accordance with the entity attributes. The original trust on both communication parties is built on both sides’ trust attributes. To make proper dynamic trust decisions, trust management must rely on context information when assigning weights to each property [15]. In the literature, there are various models of trust aggregation including the belief theory, fuzzy logic, Bayesian inference, weighted sum, and regression analysis [8].

### 2.5. Trust Update

This component decides when to update the values of trust. The updating of the trust information occurs periodically (time-driven) by applying a trust aggregation or after a transaction or event affects the QoS (event-driven) [8].

## 3. Related Works

Adopting trust management solutions is one of the promising trends that addresses the challenges raised by suspicious IoT devices and services. For instance, a study in [16] suggested a protocol for trust management consisting of three variables: cooperativeness, honesty, and community interests. Using this protocol, it was possible to create new nodes with the intent of establishing trust relationships with other nodes and endure in unsafe environments. Another study in [17] integrated two models (subject and object) to make a reliable system in regard to the objects’ performance. In the first model, each node calculated its friends based on its experiences and the friends’ thoughts in common with the potential providers to adapt behavior dynamically. In the other model, each node’s data was assigned and stored using a Distributed Hash Table structure; therefore, any node can utilize similar data.

A further study by [18] developed a trust propagation model for IoT services. The model depended on dispersed collaborative filtering to arrive at the feedback by utilizing social contact, similarity ratings of friendship, and interest relationships while using community as the filter. Both studies by [3,19] introduced fuzzy logic-based trust assessment methods. The first study used the Bio-inspired Energy Efficient-Cluster (BEE-C) protocol and fuzzy logic to compute the trust of the nodes. The value of the trust was compared to the threshold value. Trust values above the threshold were considered to be trusted nodes. Likewise, the trust value below the threshold value was defined as a non-trusted node and was eliminated. In contrast, the other study used fuzzy to solve the network traffic that influences energy dissipation through the data transmitted by sensor nodes. The scheme implemented decision-making to authenticate the sensor nodes of the network to perform a trusted aggregator.

Moreover, the study by [14] designed a simple trust management model founded on entropy and the Bayesian principle. The Bayesian principle is used to compute the value of the trust for direct nodes and periodically updated. Similarly, entropy theory distributes weights to various values of trust that can enhance the issues caused by subjectively distributing weights and also improve a model’s adaptability. Similarly, a study by [13] developed a distributed trust management model in IoT. The model’s goal was to detect malicious node activity and avoid potential on-off attacks on a multi-service IoT. Three phases of the model are contained, which are neighbor discovery, service request, and trust computation.

Furthermore, a study by [12] suggested an IoT trust and reputation-based recommendation method using a probabilistic neural network (PNN). It was performed on IoT edge devices to distinguish between untrustworthy and trustworthy nodes. The model solved the initial value trust issue in IoT environments by forecasting ratings based on the attributes for new devices and learning over time. Another study by [20] suggested a Central Trust management framework for the Internet of Things (CTM-IoT) to provide trustworthy information exchange across IoT devices. The concept included a super-node that served as a centralized trust manager. The trust information of all master nodes and cluster nodes was stored in the central repository by the super-node. The super-node was also in charge of monitoring different activities, such as network traffic and trust management, across all IoT devices. Additionally, the super-node contained a repository in which all master node trust values and addresses were kept. The repository acted as a routing table, recording trustworthy information as well as the network structure, and controlled all devices in the CTM-IoT framework, determining which devices must join which cluster.

Besides, [7] presented a computational trust model for IoT services based on machine learning techniques. This model used two techniques: (i) k-means for clustering and labeling tasks, such as identifying the number of clusters and initial centroid positions; and (ii) support vector machine (SVM) for classification tasks, such as identifying the boundaries of trustworthy and untrustworthy interaction. Similarly, a study in [21] suggested smart algorithms to manage IoT trust. The first algorithm suggested a new clustering method by calculating memory boundary trust value limits for each cluster while the second algorithm established conditions under which a cluster node in IoT trust management can be changed to a specified new master node. The third algorithm is used to address the bad-mouthed attacks. The fourth algorithm proposed methods by which master nodes track trust values for cluster nodes and attempt to shift some cluster nodes away.

A further study [22] proposed a fuzzy logic-based protocol for detecting on-off attacks, contradicting behavior attacks, and other bad nodes. This protocol allowed nodes to transfer securely from one cluster to another. Furthermore, for secure message encryption, it employed a messaging system similar to serial transmission. Additionally, the protocol utilized fuzzy logic to identify bad nodes and limit their untrusted role of making erroneous recommendations regarding nodes in the network.

A study [11] developed a model that utilizes various parameters, such as the device ownership trust, device security, and level of security in a device, to determine the trust level based on the fuzzy logic model. The fuzzy logic model was used to assess the degree of trust with using the threshold selected by users. IoT service users can also play an active role in the selection process of their trusted nodes tasked with collecting their data when the trust level is higher than the threshold. Moreover, the research by [23] suggested a trust assessment model using multi-layer perceptron (MLP). This model allowed the types of trust-related attacks carried out by malicious nodes to be detected and separated from the network to achieve a secure environment.

Another study by [24] developed a smart device selective recommendation method that utilizes a dynamic black-and-white list. The aim of this method is to eliminate the problem faced when selecting participants in order to improve the quality of services offered by edge computing systems utilizing IoT in a smart city. Game theory was introduced to qualitatively analyze the stability and validity of the proposed trust management system. In addition, Lyapunov theory was used to verify results obtained from game theory. A recent study by [25] proposed a trust framework using a neural network. This framework considered many perspectives (e.g., owner, device, and service) and each perspective considered certain attributes (e.g., social and locality for the owner, reputation of the device, and reliability of the service).

Similarly, a study in [26] developed an approach for trust management in social IoT devices. The approach contained three main stages: (1) trust composition stage—at this stage, various attributes are chosen as per the attack context. For the trust computation process, the trustee node was selected from the set of nodes based on trust attributes; (2) aggregation stage, through this stage, the trust score was calculated based on the artificial neural network (ANN) algorithm; and finally (3) the update stage, the time-driven model was used to update the trust score periodically. A study conducted by [27] recommended a dynamic trust management mechanism in wireless sensor networks (WSNs). Firstly, the node’s direct trust value is determined by evaluating its performance from interaction with regional information. After, the comprehensive trust value is calculated using the energy evaluation and trust recommendation value of other nodes with a high trust level. Finally, the node management and reliability of nodes are periodically updated.

Recently, a study by [28] suggested an information entropy-based trust evaluation approach to solve the issue of trust in the power distribution of communication terminals in Internet of Things. First, the model estimated the direct trust value based on the reputation of an exponential distribution, and then, the forgetting factor and sliding window updated the direct trust value. Uncertainties in the direct trust value were assessed, and the indirect trust value was added to compensate for inaccuracies that arise from direct trust judgment. In addition, the indirect and direct trust value were assessed completely to enhance the judgment accuracy.

Table 1 summarizes the related current techniques used for trust management in IoT devices and services based on the design components for each approach.

To conclude, managing trust is a critical problem that is seen as a significant challenge for IoT devices and services. Several solutions have been proposed in the literature as discussed earlier in this section. However, some serious research gaps are still unsolved, which are summarized in the following sections.

### 3.1. Diversity in Components of Trust Models

Studies have provided clear evidence that trust is a very complex concept and has various meanings, since they have addressed the trust issues depending on the author’s view. As can be seen in Table 1, many studies have used a variety of components while developing trust models. Therefore, the components of design trust management differ depending on the study.

In the trust composition component, the existing studies calculated this component using either quality of service (QoS) (which is done by utilizing some trust properties, such as competence, reliability, task completion capability, and cooperativeness, to measure the value of the trust [8]) or social trust (this is done by utilizing trust properties, such as honesty, centrality, intimacy, privacy, and connectivity, to measure trust values [8]). Thus, selection of the optimal components can be a challenge for managing trust. Most studies have evaluated trust management models according to the risk and logic only and ignored the composition components, which play a significant role in assessing the relationships between entities or between behaviors [29].

In both trust formation and propagation, most studies have relied on more than one attribute to measure trust. Multiple attributes make the model more accurate because the evaluation will depend on more than one feature. In addition, many studies in the literature relied on the use of a distributed model that gives trust values to the node itself without the need for an intermediate central node. Therefore, the use of distributed models is better, because the process of assigning the trust value to each node is faster, and in the case of an unauthorized attack, it reduces the risk of infection for the rest of the nodes in the system.

In terms of the aggregation component, most existing studies used both direct and indirect trust to obtain the information. However, indirect trust may cause many problems, such as incorrect recommendations and high computational capacity and time need to assign trust values, in contrast to direct trust. Besides, most studies have focused on static aggregation components, which may not be effective with the dynamic nature of IoT. Although the studies have worked on machine learning algorithms that may improve aggregation processes, they focused on the dynamics of the aggregation process itself and assigning weights manually, as is the case in fuzzy logic, which depends entirely on human experience and knowledge. Therefore, employing dynamic aggregation with dynamic assigning of weights helps to enhance data efficiency and accuracy [30].

In terms of the trust update component, updating the trust is important as it identifies changes to the node after a specific event or time. Most of the studies either ignored this component or depended on the time-driven approach while designing their models. Few studies adopted the event-driven approach. However, updating the trust based on the event is essential for some reasons, such that after any event, the trust values of the node may increase or decrease according to its behavior. Therefore, it is illogical to rely on a specific time to measure the trust of nodes that may be infected from the previous event.

### 3.2. Shortcomings of Techniques Used

The attackers mostly use untrusted entities to manipulate their behaviors and act as trustworthy entities; identifying these misbehaviors is essential. Existing studies have focused on solving a specific type of malicious behaviors, but an advanced attacker may choose a sophisticated strategy to act maliciously. Consequently, most existing research and development efforts in the domain of trust management are centered around applying statistical models or machine learning techniques, such as in [3,7,14,22,23,27]. These techniques have several drawbacks, including ineffectiveness when dealing with big data and continuously changing behaviors, high memory utilization, and difficulty in quantifying uncertainty for untrusted behaviors. Consequently, deep learning approaches may become an excellent alternative for overcoming the mentioned constraints of machine learning and statistical techniques. Deep learning has found widespread use in computer vision, speech recognition, robotics, and misbehavior detection, among a variety of other fields [31]. Deep learning offers several benefits over machine learning and statistical techniques: (1) deep learning can match complicated nonlinear connections between variables due to the usage of numerous hidden layers inside a neural network topology, (2) it is also especially well adapted to coping with “big data” issues, and (3) it is able to teach IoT devices complex behavioral patterns more successfully than machine learning and statistical techniques [31].

## 4. Proposed Model

**Figure 3:**
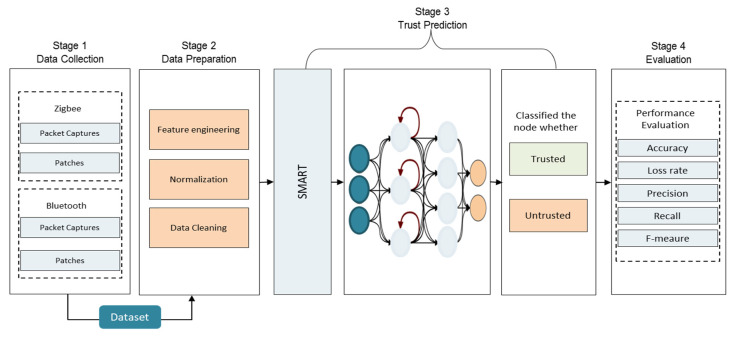
Proposed Model.

The proposed model is divided into four main stages: data collection, data preparation, trust prediction, and evaluation. Figure 3 depicts the architecture of the proposed model.

### 4.1. Data Collection Stage

This stage collects the data to test the model in the next stages. This study uses packet captures and patches proposed by [32]. The data is collected from IoT devices’ activity utilized to monitor smart homes for 10 days. IP (including Ethernet, Wi-Fi, and PPP), Bluetooth, Z-Wave, RF869, and ZigBee protocols are installed. Table 2 and Table 3 show the details of the devices and the number of captures and batches. For packet captures, it contains the information about the source and destination addresses, timestamp, data and packet length, the destination PAN id, and data. In the patches, it contains the information about the source and destination addresses, timestamp for the start and end, duration, packets number, and size of the packet.

### 4.2. Data Preparation Stage

During this stage, many sub-stages are used for data preparation, such as feature engineering, normalization, and cleaning.

#### 4.2.1. Feature Engineering

The primary goal of feature engineering is to create or extract features from existing data [33]. Therefore, at this sub-stage, some of the existing features are used to create additional features (e.g., packet loss, delay, and throughput). The following definitions and equations are according to [34].

*Packet Loss—*The failure of packets to reach their destination is referred to as *packet loss*. The value of *packet loss* can be calculated using Equation (1):

(1) $$Packet\;Loss=\frac{Packet\;sent-Packet\;recived}{Packet\;sent}\times 100$$

*Delay—*The latency caused by *transmission* from one point to another, which becomes the goal, is known as a *delay*. Equation (2) is used to calculate the delay:

(2) Delay=propagation delay+transmission delay+queuing delay+processing delay

*Propagation Delay—*The amount of time it takes for a bit to travel from the source to its destination. The *propagation delay* is computed by dividing the *distance* by the *propagation speed*, as shown in Equation (3):

(3) $$Propagation\;delay=\frac{distance\;}{Propagation\;Speed}\;$$

where the *distance* is the average *packet* size * 1000 and the *propagation speed* is the constant value (=3 × $10^{8}$ m/s).

*Transmission Delay—*The amount of time it takes to send a *packet* from the source to the *transmission medium*, as shown in Equation (4):

(4) $$Transmission\;delay=\frac{length\;of\;packets\;}{bandwidth}\;$$

where *bandwidth* represents the maximum number of *packets*.

Queuing Delay—This delay is caused by the time needed for routers to handle packet transmission queues across the network.

Processing Delay—The time it takes a network device to see the route, update the header, and switch tasks is referred to as processing delay.

*Throughput* refers to the actual *bandwidth* that was measured at a specific time and under specific network conditions to transfer files of a specific size. The total speed of data sent to all terminals in a network is known as throughput, which can be calculated using Equation (5):

(5) $$Throughput=\frac{\sum Packet\;sent\left(bits\right)}{Time\;of\;data\;deilvary\;\left(s\right)\;}\times 100$$

#### 4.2.2. Normalization

In this process, the features are scaled to values ranging from 0 to 1 to produce an accurate result. This step is necessary to transform the numeric column values in the dataset; therefore, it may be used on a common scale without distorting the variation in value ranges or losing data [35]. The normalization is performed using Equation (6):

(6) $$z_{i}=\frac{x_{i}-\min\left(x\right)}{\max\left(x\right)-\min\left(x\right)\;}$$

where $x_{i}$ is the dataset’s *i*th value, min(*x*) is the dataset’s minimum value, and max(*x*) is the dataset’s maximum value.

#### 4.2.3. Data Cleaning

This sub-stage aims to clean the data by assuring the validity of dataset samples, such as removing the null and negative values in records.

### 4.3. Trust Prediction Stage

The trust prediction stage is divided into two sub-stages: the calculation of the trust value and misbehaving detection. In the trust value calculation sub-stage, the simple multi-attribute rating technique (SMART) is used, which determines the value of the trust based on the node information extracted in the previous stage (data preparation). In the misbehaving detection sub-stage, the long short-term memory (LSTM) technique is used for classification/prediction tasks, which is known as an excellent technique for identifying changes in behavior. During this sub-stage, the learned model classifies new unknown data (included in the test set) that the model has never seen before to assess the learned model’s capabilities (initially, the detective ability of the model is evaluated and, if it is acceptable, then the learned model can be used for detection). The following subsections detail those two sub-stages.

#### 4.3.1. Trust Value Calculation

**Figure 4:**
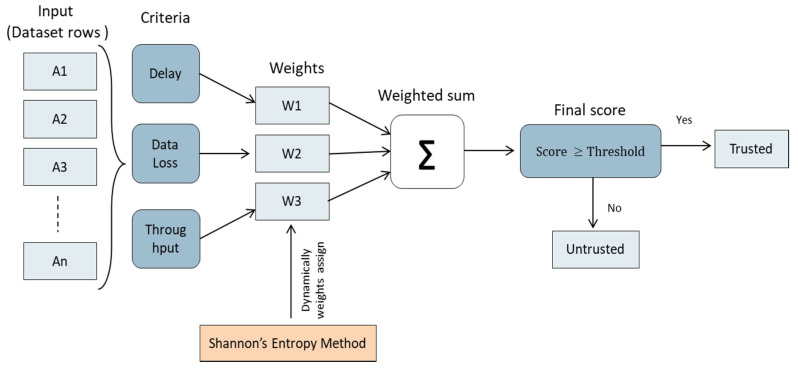
Trust value calculation using the SMART technique.

In this sub-stage, the data is identified as trusted or untrusted using the SMART technique. The SMART technique is a method used for solving multi-criteria decision-making (MCDM) issues. It is founded on the concept that each alternative is composed of a number of criteria with values, and each criterion has a weight that indicates its relevance in comparison to other criteria [36,37]. Figure 4 shows how the SMART calculates the trust value.

Step 1: Decision context and structuring includes defining the alternatives and determining the number of criteria that will be utilized.

Step 2: Analysis, which includes the following:

1. Determining the criteria weights for each criterion using the 1 to 100 scale for the criterion using Shannon’s entropy method, which is a well-known approach for determining weights for an MADM issue (e.g., static weight assign) [38]. Shannon’s entropy method is designed as an objective method of allocating weights based on the decision matrix without impacting the decision maker’s preference [39,40].

In this study, a combination of the SMART and Shannon’s entropy methods is used to calculate weights dynamically based on the specified criteria. The Shannon’s entropy (*E_j_*) can be calculated using Equations (7)–(9). Suppose *k_j_* (*j* = 1, 2, 3...) includes various alternatives and *k_i_* (*i* = 1, 2, 3...) represents the criteria inside these alternatives. The *i*th criteria value in the *j*th alternative is then indicated by *k_ij_*, and the weight evaluation procedure is created on this basis. Because the dimensions of the various alternatives throughout the evaluation are not similar, these factors should be standardized using Equation (7):

(7) $$Rij=\frac{k_{ij}}{\sum_{i=1}^{m}\sum_{i=1}^{n}k_{ij}}$$

where *R_ij_* denotes the specific gravity per *k_ij_* and *m* denotes the number of criteria. Then, the entropy for each factor alternative *E_j_* is calculated using Equation (8):

(8) $$E_{j}=\left[\frac{-1}{\ln\left(m\right)\;}\right]\;\;\sum_{i=1}^{m}\;\left[R_{ij}\ln\left(R_{ij}\right)\right]$$

where *m* is the number of standardized assessment possibilities in the matrix and *ij* is the number of criteria.

(9) *D_j_ =* 1 − *E_j_*

where *D_j_* is the diversity criterion.

2. Normalizing each criterion by dividing the number of weighted criteria by the number of weights, using Equation (10):

(10) $$\mathrm{Wj}=\frac{D_{j}}{\sum_{j=1}^{k}D_{j}}$$

where $D_{j}$ is the weight value of the criteria, $\overset{k}{\underset{j=0}{\sum }}D_{j}$ is the total weight of all criteria, and j is the number of possibilities from 1 to *k*.

3. Providing a value for each criterion parameter for each alternative.

Step 3: Decision, which involves the following:

1. Determining the utility value to transform the value of each criterion‘s criteria into the value of the raw data criteria. Equation (11) is used to calculate the utility value:

(11) $$u_{i}\left(a_{i}\right)=\frac{c_{out}-c_{min}}{c_{max}-c_{min}}$$

where $u_{i}\left(a_{i}\right)$ denotes the utility value of the criterion to *i* for the criterion to *j*, $c_{max}$ is the greatest criterion value, $c_{min}$ is the lowest criterion value, and $c_{out}$ is the criteria value of *i*. The significance of these values is shown in Equation (12):

(12) $$c_{out\;}i=u_{i}\left(a_{i}\right),\;\;1=0;\;2=0.5;\;3=1$$

Equation (11) is used to determine the value of the utility to convert the value of the criterion to *i* one of the criteria to *i*. The computation then yields the following results:

- If the criteria value ($c_{out\;}$) = 3, then $u_{i}\left(a_{i}\right)=$ $\frac{3-1}{3-1}$ = 1;
- If the criteria value ($c_{out\;}$) = 2, then $u_{i}\left(a_{i}\right)=$ $\frac{2-1}{3-1}$ = 0.5;
- If the criteria value ($c_{out\;}$) = 1, then $\;u_{i}\left(a_{i}\right)=$ $\frac{1-1}{3-1}$ = 0.

2. Determining the final value of each criterion by shifting the values obtained from the normalized value of the raw data criteria with weight normalized value criteria, using Equation (13):

(13) $$u\left(a_{i}\right)=\sum_{j=1}^{n}w_{j\;}u_{i\;\;},\;\mathrm{where}\;i=1,2,\ldots \ldots ,n$$

3. Calculating the dynamic threshold (DT) using the mean absolute error (MAE). MAE is a metric that measures how close predictions are to actual results. MAE is employed because it gives a straightforward means of determining the degree of significance of errors [41]. It is commonly used in the security field to quantify errors depending on the problem. In particular, it is used in trust management to determine the threshold or ground value, as defined in [42,43]. In this paper, the MAE is used for DT, as defined in Equation (14):

(14) $$\mathrm{Dynamic}\;\mathrm{Threshold}\;(\mathrm{DT})=\frac{\sum_{i=1}^{n}\left|u\left(a_{i}\right)-\bar{u\left(a_{i}\right)}\right|}{n}$$

where $u\left(a_{i}\right)$ is the value of the trust, $\bar{u\left(a_{i}\right)}$ is the predicted trust value, and *n* is the total number of samples.

4. Comparing the value of trust with the DT that was obtained using Equation (15), if the value of trust is greater or equal to the DT value, this means the device is trusted; otherwise, it is untrusted:

(15) $$Trust\;Score=\{\begin{array}{c} \;\;u\left(a_{i}\right)<\mathrm{DT},\;\;Untrused\\ u\left(a_{i}\right)\geq \mathrm{DT},\;\;Trusted\; \end{array}$$

#### 4.3.2. Misbehaving Detection

In this sub-stage, the long short-term memory (LSTM), a deep learning technique that has recently sparked the interest of the research community, is used. LSTM has produced excellent results when applied to complicated issues, such as the translation of languages, text generation, and automatic captioning of images, among other applications [44]. This technique has been widely used in recent years to overcome security issues, such as in [45,46,47]. For this reason, this study uses LSTM to detect malicious behaviors that may indicate trust violation issues.

### 4.4. Evaluation Stage

This stage includes a detailed experimental assessment of the proposed model. There are several metrics for various algorithms, and these metrics have been devised to evaluate the effectiveness and efficiency of the models. The false positive (FP), false negative (FN), true positive (TP), and true negative (TN), as well as their relationships, are parameters often used by trust management researchers to evaluate the performance of various techniques [48]. The definitions of these parameters are as follows:

True Positive (TP) indicates the part of the untrustworthy entities properly identified as untrusted.

True Negative (TN) indicates the part of the trustworthy entities properly identified as trusted.

False Positive (FP) represents the part of the trustworthy entities wrongly identified as untrustworthy.

False Negative (FN) represents the part of the untrustworthy entities wrongly identified as trustworthy.

This study employs five different metrics based on these parameters, which include accuracy, loss rate, precision, recall, and F-measure, to measure how well the model performs.

Accuracy is defined as the degree of agreement between the actual measurement and the absolute measurement. Accuracy is one of the most widely used metrics of classification performance, and it is described as the ratio of properly categorized samples to the total number of samples [49], and is calculated as Equation (16):

(16) $$\mathrm{Accuracy}=\frac{\mathrm{TP}+\mathrm{TN}}{\mathrm{TP}+\mathrm{TN}+\mathrm{FP}+\mathrm{FN}}$$

Loss Rate is a function that quantifies the difference between the actual and predicted output during training to accelerate the learning process. It is also used to evaluate model performance and minimize error [45]. Equation (17) is used to compute the loss rate:

(17) $$\mathrm{Loss}=-\;Y\times \mathrm{Log}\left(Y_{\mathrm{Pred}}\right)-\left(1-Y\right)\times \;\mathrm{Log}\left(1-Y_{\mathrm{Pred}}\right)$$

Precision refers to the classification model’s ability to retrieve just data points from a certain class. It is defined as the number of correct samples recovered divided by the total number of samples retrieved [50], and is shown in Equation (18):

(18) $$\mathrm{Precision}=\frac{\mathrm{TP}}{\mathrm{TP}+\mathrm{FP}}$$

*Recall* is the capacity of a classification model to recognize all data points in a relevant class. It calculates the number of accurate samples retrieved divided by the total number of correct samples [50], and is defined as in Equation (19):

(19) $$Recall=\frac{\mathrm{TP}}{\mathrm{TP}+\mathrm{FN}}$$

*F-Measure* is another measure derived from *precision* and *recall* that represents the behavior of both measures [51]. It is computed as shown in Equation (20):

(20) $$F-\;Measure=2\times \frac{precision\times recall\;}{precision+recall}$$

The implementation of the methodology of this study relies on several trust components, as shown in Table 4. For trust composition and formation, it is done by using multiple features to measure the QoS (*packet loss*, *delay*, and *throughput*). For trust propagation, the model uses decentralized methods to propagate the trust to reduce the loss in case of an attack infection. For the trust aggregation, the model uses both dynamic calculations of processes and weights using SMART and entropy. Finally, for the trust update, the model takes advantage of the benefit of the LSTM at detecting the changes after any event; therefore, it is used for updating purposes.

## 5. Experimental Investigation

The setup of the model and the dataset description is presented in the following subsections.

### 5.1. Model Setup

This experiment was carried out on Google CoLab with the help of Python library packages, such as Pandas, Numpy, Scikitlearn, Matplotlib, and Keras, to calculate the trust value and perform the preprocessing task. The misbehaving detection model was created with LSTM cells, drop out, and dense output layers. Table 5 describes the layers and the values of the parameters used. The model was run using 50 and 100 epochs and a batch size of 72. Furthermore, the model employed the Rectified Linear Unit (ReLu) and sigmoid activation functions and Adam optimizer.

### 5.2. Dataset Description

The dataset was split into training and testing sets with a ratio of 70:30, respectively. To avoid over fitting and under fitting, the data was randomly divided several times until it was verified that the testing set represented behaviors that were unseen before.

## 6. Results and Analysis

This section presents the dataset collection and visualization. Additionally, this section discusses the trust prediction stage experimental results and results analysis. Through this sub-section, the value of trust and misbehavior detection are discussed.

### 6.1. Dataset Collection and Visualization

**Figure 5:**
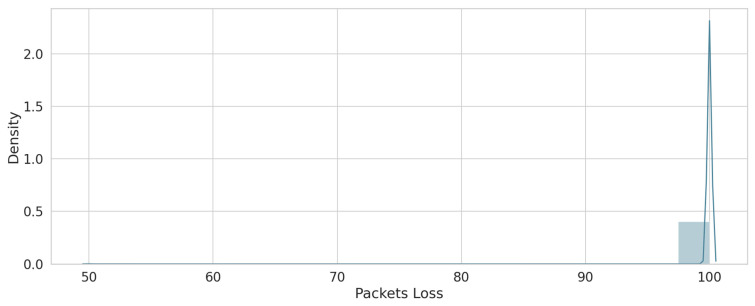
Packets loss sample.

**Figure 6:**
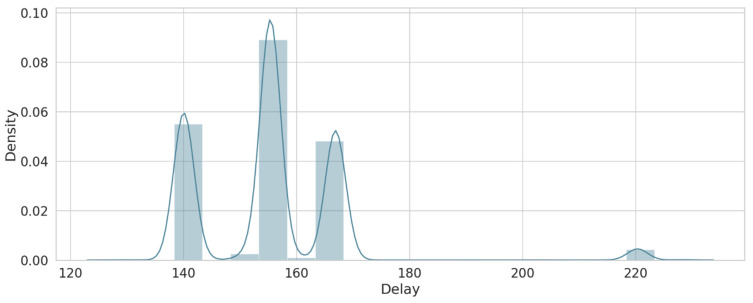
Delay sample.

**Figure 7:**
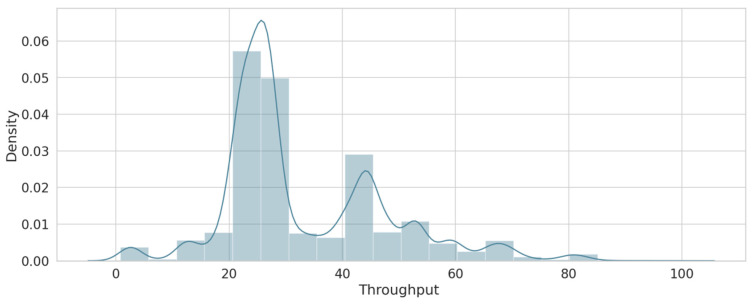
Throughput sample.

As a result of the feature engineering process described in Section 4, three features were obtained, including packet loss, delay, and throughput. Table 6 describes the ranges of each feature, which vary from good, medium, to poor based on the study by [34]. For packet loss, this feature was calculated to detect any changes that may affect the availability of the services and guarantee their reliability. Figure 5 shows the density of loss values for the overall dataset. The delay feature was calculated to test the performance of the network (when the value of delay is high, it will decrease network performance). Figure 6 shows that the delay has varying values, but most of these density values are between 140 and 170. The throughput feature was calculated as forecasted to match the demands of the current network’s services. Figure 7 shows the density of the throughput values in the dataset. These features were used as input for the next stage (the trust value prediction stage).

### 6.2. Trust Prediction Results

In this sub-section, the results of the trust value calculation and misbehaving detection are presented.

#### Trust Value Calculation Results

Step 1: Decision context and structuring: for the dataset used in this study, IoT devices represent the alternatives. Additionally, this study determined whether the device is trusted or not by investigating the three criteria (packet loss, delay, and throughput), as shown in Table 7. Each criterion has a range that indicates how well this device does. These ranges vary from poor, medium, to good, which are denoted as 1, 2, and 3, as shown in Table 8. The range helps to rearrange the values of criteria in the dataset. As seen in Table 9, in C1, which represents the packet loss, if the value in the data is less than 3%, it means the data is good and will be represented by a value of 3. Otherwise, if the value in the data is between 3% and 15%, it means the data is medium and will be represented by a value of 2. Moreover, if the value is more than 15%, the data is indicated as poor and will be represented by a value of 1. C2 and C3 also use the same analogy as C1.

Step 2: Analysis: the weights of each criterion (packet loss, delay, and throughput) were determined using Shannon’s entropy method. As the first step, rescale was done, which makes the data lie in the same range using normalization of the decision matrix (divided by the sum of each column) using Equation (7). Then, the entropy value was calculated using Equations (8) and (9). Weights vary dynamically with different data or sample sizes. The entropy method was applied to the different sizes of the data set. The results showed unobserved differences in all sample sizes, where it achieved a packet loss weight of 0.005340, a throughput weight of 0.493564, and a delay of 0.501096 using 25% of the sample size. For the 50% and 100% sample sizes, the results are shown in Table 10.

**Figure 8:**
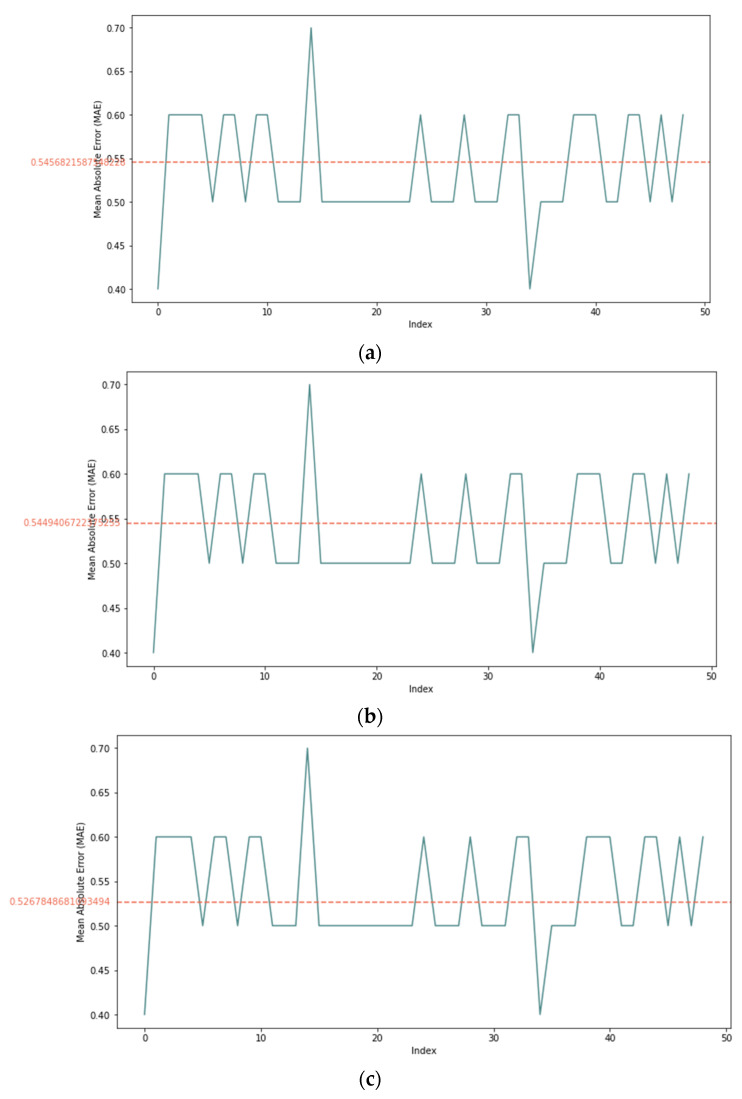
DT results for each sample: (**a**) 25% sample size, (**b**) 50% sample size, and (**c**) 100% sample size.

Step 3: Decision: in this step, the value of the trust was calculated and compared with DT. After obtaining the weights of the criteria values, the SMART method was used to aggregate the utility value by using Equations (12) and (13). Finally, the score of the trust was calculated using Equation (14) and compared with the threshold using Equation (15). Figure 8 shows the DT for each sample of data. Any values above the DT were considered as trusted while any values below the DT were considered as untrusted.

For further clarification, let us take the 100% size of the dataset (used in this study). We determined whether the device is trusted or not by investigating the three criteria (packet loss, delay, and throughput) using weighted values (0. 003108, 0.467722, and 0.529170). Let us consider an IoT device as an alternative with the following parameters: packet loss = 0%, delay = 150 ms, and throughput =75%. This means the packet loss is good, the delay is also good, and the throughput is medium. As indicated in Table 8, the values of the criteria will be 3, 3, and 2. Based on Equation (9), the values of utility are:

- The value criteria ($c_{out\;}$) = 3, then $u_{i}\left(a_{i}\right)=$ $\frac{3-1}{3-1}$ = 1;
- The value criteria ($c_{out\;}$) = 3, then $u_{i}\left(a_{i}\right)=$ $\frac{3-1}{3-1}$ = 1;
- The value criteria ($c_{out\;}$) = 2, then $u_{i}\left(a_{i}\right)=$ $\frac{2-1}{3-1}$ = 0.5.

As the last step, the score was calculated using Equation (13). Therefore, the value 0.70469100 is greater than DT, which means the device is (trusted). The same mechanism was used on the whole data set to calculate the value of the trust, which was used as input for the next step (misbehaving detection).

### 6.3. Misbehaving Detection Results

**Figure 9:**
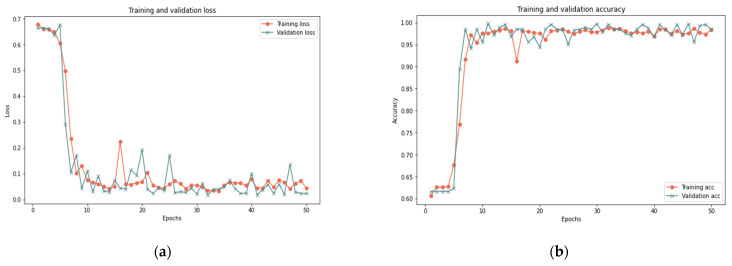

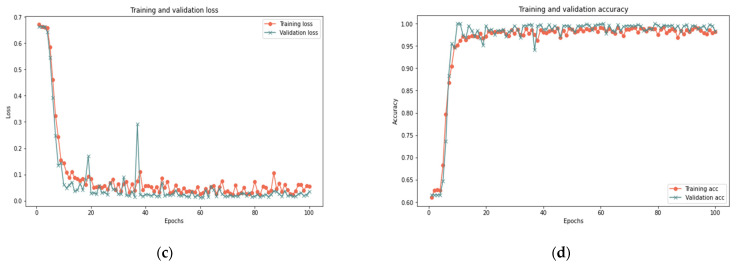
Results of the 25% sample size: (**a**) loss of 50 iterations, (**b**) accuracy of 50 iterations, (**c**) loss of 100 iterations, (**d**) accuracy of 100 iterations.

**Figure 10:**
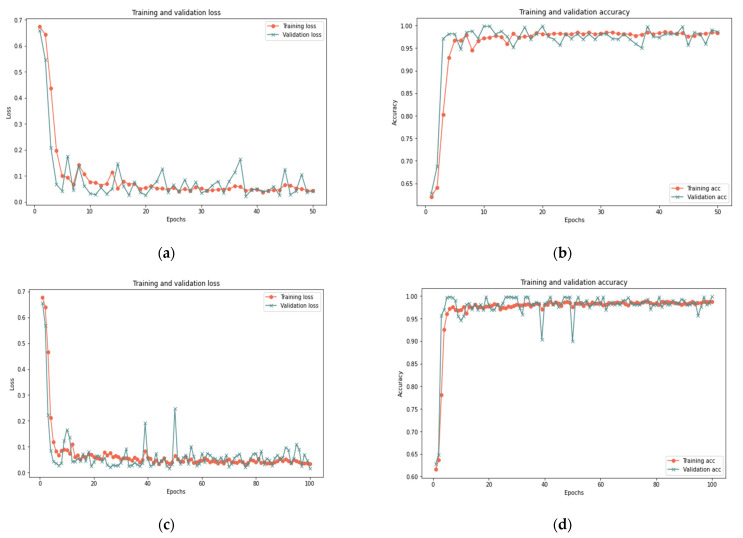
Results of the 50% sample size: (**a**) loss of 50 iterations, (**b**) accuracy of 50 iterations, (**c**) loss of 100 iterations, (**d**) accuracy of 100 iterations.

**Figure 11:**
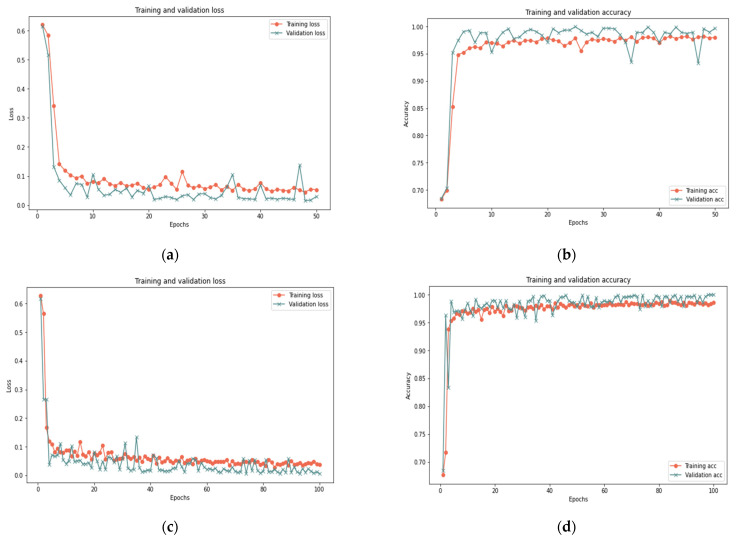
Results of the 100% sample size: (**a**) loss of 50 iterations, (**b**) accuracy of 50 iterations, (**c**) loss of 100 iterations, (**d**) accuracy of 100 iterations.

In this sub-section, the proposed model is evaluated on dataset samples of various sizes (25%, 50%, and 100%) and a different number of iterations (50 and 100) to measure the robustness of the model under various dataset settings. Table 11 reports that the proposed model for the 100% sample size shows perfect results using 50 iterations, as it obtained 99.33% accuracy, 0.023 loss rate, 100% recall, 99.26% precision, and 99.63% F-measure in 356 s. For the 100 iterations, the model shows some improvement since it obtained 99.37% accuracy, 0.018 loss rate, 100% recall, 99.93% precision, and 99.65% F-measure in 420 s. In the 25% and 50% sample sizes, similar results are reported for 100 and 50 iterations with a slight difference in execution time. Figure 9, Figure 10 and Figure 11 show the accuracy and the loss rate for each iteration with different samples size.

Based on the results presented in Section 5.1 and Section 5.2, the model effectively identified behavior deviation using LSTM cells, where it produced good results for various data samples size. Changing the sample size in the experiment proved the robustness of the proposed model in dealing with different conditions. The slight difference in the results is normal and reflects the ability of the proposed model to deal with small or big data samples. The model showed an improvement in the results with the increase of the test sample. Therefore, it is clear that there is a relationship between the sample size and the model’s ability to deal with big data. In addition, increasing the number of iterations has little impact on the results in terms of accuracy, recall, precision, and F-measure but a great impact on the time and loss rate. As illustrated in Figure 9, Figure 10 and Figure 11, the results demonstrated a substantial relation between the loss rate and the number of iterations, implying that the model is learned with each iteration. Furthermore, the time and number of iterations have a positive connection; as the number of iterations increases, so does the time. There is a negative relation between accuracy, recall, precision, F-measure, and loss rates: as accuracy, recall, precision, and F-measure increase, loss rate decreases. Consequently, this proposed model can identify suspicious activities and take appropriate actions, such as helping in redirecting IoT functionality to trustworthy zones upon identifying untrusted entities.

## 7. Comparison with Existing Approaches

Using the same dataset samples, the proposed misbehaving detection model performance is benchmarked against the most common and recent machine and deep learning-based approaches in the following subsections.

### 7.1. Comparison with Existing Deep Leaning Techniques

A comparison was performed between LSTM and the MLP, and the ANN, which were used in the literature as deep learning architectures (MLP in [26] and ANN in [25]) based on the model setup in Section 5.1. Theoretically, to select a suitable model, it is important to take the dataset patterns into consideration because fitting the data with the models is a condition for ensuring its effective performance. Deep learning is often used for solving big data issues, but each model is designed for specific tasks depending on the data used [31]. For example, the LSTM is usually used for time sequences and difficult learning tasks, such as prediction tasks, detecting changes in behaviors [44], machine translation [52], and handwriting generation [53]. MLP is mostly used for image processing tasks [54], and ANN is usually used for image processing, character recognition [55], and forecasting [56]. In this study, the data is IoT devices’ activities, which means the type of data is behavioral patterns. Therefore, the model should be able to deal with behaviors and their changes. This proves that the LSTM model will be more compatible for identifying the changes in trusted and untrusted behaviors than other proposed models based on the ANN and MLP algorithms.

**Figure 12:**
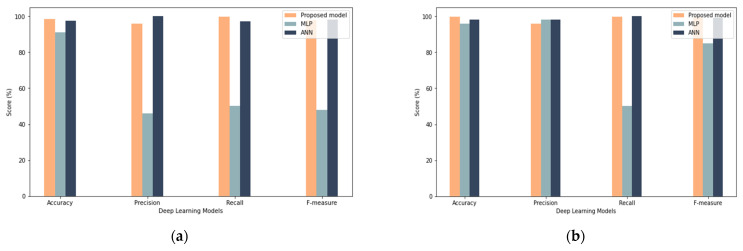
Comparison with Deep Learning Results (**a**) shows results for 25% sample size with 50 iterations (**b**) show results for 25% sample size with 100 iterations.

**Figure 13:**
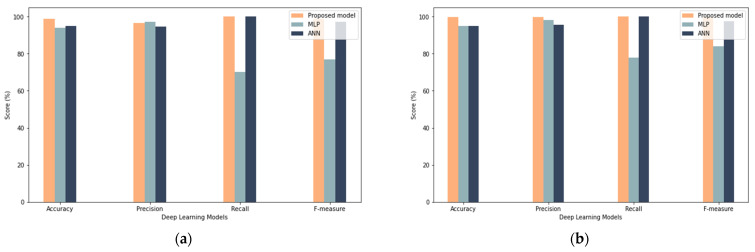
Comparison with deep learning results: (**a**) results for the 50% sample size with 50 iterations, (**b**) results for the 50% sample size with 100 iterations.

**Figure 14:**
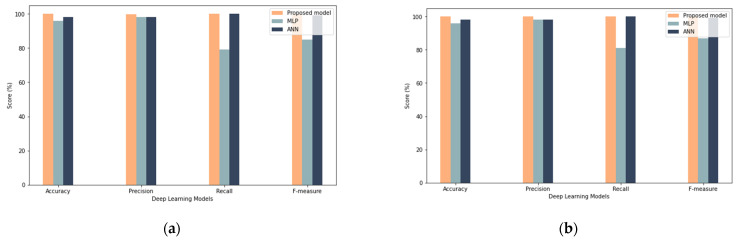
Comparison with deep learning results: (**a**) results for the 100% sample size with 50 iterations, (**b**) results for the 100% sample size with 100 iterations.

From a practical perspective, the proposed model obtained good results among all different sample sizes, which proves that the model has enhanced robustness by obtaining close results with different sample sizes, as shown in Figure 12, Figure 13 and Figure 14. Oppositely, the ANN and MLP models showed a discrepancy between the results (such as in recall, precision, and F-measure) with the change in the size of the samples. In summary, the LSTM is an excellent choice for modeling sequential data and is thus used to understand complicated activity dynamics. The reason for this is that stacked LSTM networks may learn higher-level temporal patterns without prior knowledge of the pattern; therefore, stacked LSTM networks may be a feasible approach for modeling regular time series behavior, which can then be used to detect misbehavior.

### 7.2. Comparison with Existing Machine Leaning Techniques

**Figure 15:**
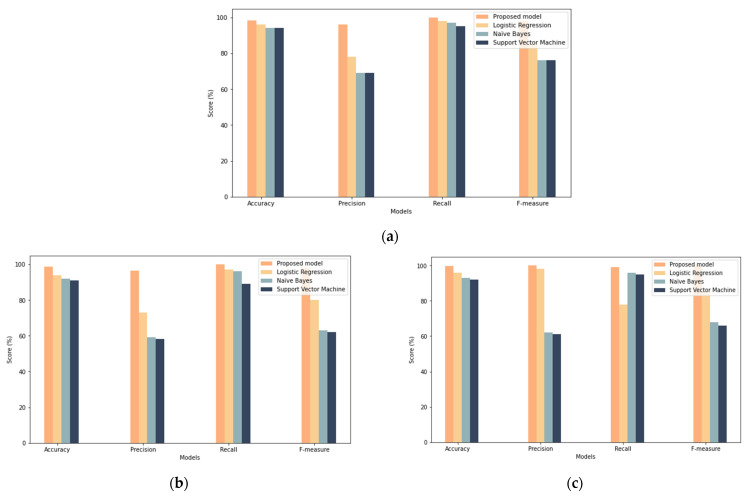
Comparison with machine learning results: (**a**) results for the 25% sample size, (**b**) results for the 50% sample size, (**c**) results for the 100% sample size.

A comparison with existing machine learning techniques was performed to evaluate their performance alongside the proposed model. The comparison was performed with different samples sizes. As shown in Figure 15, the proposed model demonstrated the best overall performance among machine learning models. In particular, it showed the highest performance in terms of accuracy, recall, precision, and F-measure compared to other models. In the case of a 25% and 50% sample size, the machine learning models gave accuracy results similar to the proposed model, but in other measurements (e.g., recall, precision, and F-measure), the proposed model outperforms them. In the case of a 100% sample size, the results of the proposed model increased, while the results of the rest of the models decreased. These results gave clear evidence that the performance of the proposed model improves with the increase in the sample size used, unlike the other models whose performance decreases with the increase in the sample size. Additionally, changing the samples gave evidence that the deep learning models are more robust, as the results indicate a more stable performance despite the different samples, which makes them excellent candidates for dealing with continuous changes in IoT devices and detecting misbehavior. In conclusion, the proposed model appears to outperform other machine learning models while these models fail to reach a significant performance. This proves that the LSTM can be adapted to deal with “big data” challenges and can teach complicated behavioral patterns of IoT devices more successfully than machine learning models.

## 8. Conclusions and Future Work

Managing trust is an issue with far-reaching consequences for an artificial society, such as IoT. In recent years, increased dependence on IoT devices and services has aggravated this issue. Traditional models are no longer effective in the age of big data and IoT devices have a dynamic nature and heterogeneity. As a result, this paper suggested a model for trust management in IoT devices and services that are based on the simple multi-attribute rating technique (SMART) and long short-term memory (LSTM). SMART was used to calculate the value of trust based on the information of the node itself, essentially decreasing the risk of attacks caused by incorrect recommendations. In addition, the LSTM was used to detect changes in behavior with high accuracy. Experimental results revealed that the proposed model achieved 99.87% and 99.76% accuracy and F-measure with 100 iterations, respectively. Finally, a comparison with the existing machine and deep learning techniques showed that the proposed model can achieve a superior performance in addressing trust-related problems of the IoT world. In future work, more features will be considered to calculate the value of the trust (e.g., energy consumption of IoT devices).

## Figures and Tables

**Table 1:** Summary of existing studies.

Approach	Components of Design Trust Management								
	Trust Composition	Trust Formation		Trust Propagation		Trust Aggregation		Trust Update	
		Single-Trust	Multi-Trust	Centralized	Distributed	Static	Dynamic	Time-Driven	Event-Driven
[16]	√	x	√	x	√	x	x	√	x
[17]	√	x	√	x	√	√	x	x	x
[18]	√	x	√	x	√	x	√	√	x
[19]	√	x	√	x	√	√	x	x	x
[14]	x	x	√	x	√	x	√	√	x
[13]	x	x	√	x	√	√	x	x	x
[3]	x	x	√	x	√	√	x	x	x
[12]	√	x	√	x	√	x	√	√	x
[20]	x	√	x	√	x	x	x	x	√
[7]	√	x	√	x	√	x	√	x	x
[21]	x	x	x	√	x	x	√	√	x
[22]	x	x	x	√	x	x	√	x	√
[11]	√	x	√	x	√	√	x	x	x
[23]	√	x	√	x	√	x	√	√	x
[24]	x	√	x	x	√	x	√	x	√
[25]	√	x	√	x	√	x	√	x	x
[26]	√	x	√	x	√	x	√	√	x
[27]	x	x	√	x	√	x	x	√	x
[28]	x	x	x	x	√	√	x	√	x

**Table 2:** Device deployment places [32].

Device Type	Protocol	Placement
Motion sensor	Zigbee	Living room
Motion sensor	Zigbee	Kitchen
Motion sensor	Zigbee	Bathroom
Motion sensor	Zigbee	Bedroom
Door sensor	Zigbee	Entrance door
Door sensor	Zigbee	Dishwasher
Weight scale	Bluetooth	Nearby the gateway
Blood pressure meter	Bluetooth	Nearby the gateway
Gateway	Bluetooth	Office
Gateway	Zigbee	Office

**Table 3:** Number of packets and patches for each protocol [32].

Protocol	Packet Captures	Patches
Zigbee	73,876	27,385
Bluetooth	541,544	22,202

**Table 4:** Trust components used in this study.

Trust Component	How It Is Applied in the Implementation Part?	Stage No.
Trust Composition	By measuring the QoS for the obtained features	2 and 3
Trust Formation	Using multi multi-features to calculate the value of the trust	3
Trust Propagation	Calculate the value of trust that means the propagation is decentralized	All model
Trust Aggregation	Using both SMART and entropy to dynamically aggregate the value of features and dynamically calculated the weight of features	3
Trust Update	By applying LSTM cells to detect the changes after an event happened	3 and 4

**Table 5:** Model setup settings.

Parameters	Value
Language	Python
Libraries	Pandas, Numpy, Scikitlearn, Matplotlib, and Keras
Train set	70%
Test set	30%
Input Layer	1
LSTM Cells	2 cells
Activation Functions	Rectified Linear Unit (ReLu), and sigmoid
Dense Layer	1
Dropout	0.20
Optimizer	Adam
Number of Epochs	50 and 100
Batch size	72

**Table 6:** Ranges of the selected features.

Range	Feature Name and Its Ranges		
	Packet Loss	Delay	Throughput
Good	Less than 3%	0–150 ms	100%
Medium	More than 15%	151–400 ms	75–50%
Poor	More than 25%	More than 400 ms	Less than 25%

**Table 7:** Alternatives with criteria.

Alternative	Criteria (C)		
	Packet Loss (C1)	Delay (C2)	Throughput (C3)
A1	0	100	103
A2	100	400	105
…	…	…	…
An	100	305	190

**Table 8:** Criteria values.

Group	Parameter Value
Poor	1
Medium	2
Good	3

**Table 9:** Value of sub criteria.

No.	Criteria (C)	Range	Value
1.	C1	1. >3%	3
		2. <3–15%	2
		3. <15–25%	1
2.	C2	1. 0–150 ms	3
		2. 151–400 ms	2
		3. >400 ms	1
3.	C3	1. >25%	3
		2. 50–75%	2
		3. 100%	1

**Table 10:** Criteria weights for each data sample.

Sample Size = 25%		
**No.**	**Criteria (C)**	$w_{j}=\;(\frac{Dj}{\sum Dj})$
1.	C1	0.005340
2.	C2	0.493564
3.	C3	0.501096
Sum		1
Sample size = 50%		
**No.**	**Criteria (C)**	$w_{j}=\;(\frac{Dj}{\sum Dj})$
1.	C1	0.003222
2.	C2	0.498422
3.	C3	0.498355
Sum		1
Sample size = 100%		
**No.**	**Criteria (C)**	$w_{j}=\;(\frac{Dj}{\sum Dj})$
1.	C1	0.003108
2.	C2	0.467722
3.	C3	0.529170
Sum		1

**Table 11:** Experimental results of the dataset with different sample sizes.

**Sample Size = 25%**						
**Iterations**	**Accuracy (%)**	**Loss Rate**	**Recall (%)**	**Precision (%)**	**F-Measure (%)**	**Time(s)**
50	98.33	0.0350	99.85	95.97	97.87	88
100	98.50	0.00223	96.70	99.38	98.02	180
**Sample Size = 50%**						
**Iterations**	**Accuracy (%)**	**Loss rate**	**Recall (%)**	**Precision (%)**	**F-Measure (%)**	**Time(s)**
50	98.62	0.0125	99.92	96.50	98.18	84
100	99.81	0.0115	99.92	99.69	99.81	185
**Sample Size = 100%**						
**Iterations**	**Accuracy (%)**	**Loss rate**	**Recall (%)**	**Precision (%)**	**F-Measure (%)**	**Time(s)**
50	99.66	0.0082	98.986	100	99.49	356
100	99.97	0.0059	100	99.92	99.96	420
